# A Reconsideration of the Self-Compassion Scale’s Total Score: Self-Compassion versus Self-Criticism

**DOI:** 10.1371/journal.pone.0132940

**Published:** 2015-07-20

**Authors:** Angélica López, Robbert Sanderman, Ans Smink, Ying Zhang, Eric van Sonderen, Adelita Ranchor, Maya J. Schroevers

**Affiliations:** 1 Department of Health Sciences, Health Psychology Section, University of Groningen, University Medical Center Groningen, Groningen, The Netherlands; 2 Department of Psychology, Health and Technology, University of Twente, Enschede, The Netherlands; 3 Department of Applied Psychology, Lingnan University, Hong Kong, China; Tilburg University, NETHERLANDS

## Abstract

The Self-Compassion Scale (SCS) is currently the only self-report instrument to measure self-compassion. The SCS is widely used despite the limited evidence for the scale’s psychometric properties, with validation studies commonly performed in college students. The current study examined the factor structure, reliability, and construct validity of the SCS in a large representative sample from the community. The study was conducted in 1,736 persons, of whom 1,643 were included in the analyses. Besides the SCS, data was collected on positive and negative indicators of psychological functioning, as well as on rumination and neuroticism. Analyses included confirmatory factor analyses (CFA), exploratory factor analyses (EFA), and correlations. CFA showed that the SCS’s proposed six-factor structure could not be replicated. EFA suggested a two-factor solution, formed by the positively and negatively formulated items respectively. Internal consistency was good for the two identified factors. The negative factor (i.e., sum score of the negatively formulated items) correlated moderately to strongly to negative affect, depressive symptoms, perceived stress, as well as to rumination and neuroticism. Compared to this negative factor, the positive factor (i.e., sum score of the positively formulated items) correlated weaker to these indicators, and relatively more strongly to positive affect. Results from this study do not justify the common use of the SCS total score as an overall indicator of self-compassion, and provide support for the idea, as also assumed by others, that it is important to make a distinction between self-compassion and self-criticism.

## Introduction

In recent years, there has been a growing research interest in the concept of self-compassion, particularly as an outcome of mindfulness-based interventions [1,2], and more recently compassion-based interventions [3–5]. The cultivation of a mindful, nonjudgmental, compassionate attitude is assumed to be a key mechanism that may explain the benefits of these interventions for improvements in psychological outcomes [6]. Self-compassion is typically assessed by the Self-Compassion Scale (SCS) [7], as this is currently the only self-report questionnaire available for measuring self-compassion. However, evidence regarding its psychometric properties is limited, particularly as it has been mostly tested in college students. Therefore, the aim of this study was to examine the psychometric properties of the SCS in a large representative sample from the general population.

(Self)-compassion can be regarded as the recognition of distress or suffering and the desire to alleviate it [4]. The definition of Neff [8] is the most commonly used within research literature. According to Neff [8], self-compassion includes three components: *self-kindness*—treating oneself with tenderness and understanding when facing suffering rather than with harshness and self-judgment—a sense of *common humanity*—seeing one’s failures as part of the human condition rather than feeling isolated—and *mindfulness*—having a balanced awareness of the present experience instead of over-identifying with painful thoughts and emotions.

There is a growing body of evidence that supports the benefits of self-compassion for subjective wellbeing. A recent meta-analysis found a large effect size for a negative relationship between self-compassion and psychological symptoms (i.e., stress, anxiety and depression), with higher levels of self-compassion being strongly associated with lower levels of symptoms [9]. Besides its association with the presence of psychological symptoms, self-compassion has been moderately positively related to the experience of positive affect [10].

Most studies on self-compassion measure the concept with the Self-Compassion Scale [7]. This 26-item questionnaire was developed based on Neff’s [8] definition of self-compassion, as described previously. Each of the three components, i.e., self-kindness, common humanity, and mindfulness, is measured by both positively and negatively formulated items. In the original study, Neff [7] found that the positively and negatively formulated items formed separate factors rather than one: self-kindness versus self-judgment, common humanity versus isolation, and mindfulness versus over-identification. As a result, Neff proposed a six-factor structure with one higher-order factor of self-compassion [7]. Thus, the SCS contains six subscales that can be added to obtain a total score of self-compassion. Most researchers use this total score as an indicator of self-compassion.

Some studies have tested by confirmatory factor analysis (CFA) the SCS’s factor structure revealing mixed findings. One study confirmed the six-factor structure with a higher-order factor of self-compassion in a sample of students [11]. Other studies replicated the six-factor structure, but could not find evidence for a higher-order factor among community samples [12,13], indicating that the six subscales cannot be summed into an overall score of self-compassion. In a recent study, however, none of the models could be replicated, neither in a sample of meditators, nor in depressed patients and adults from the community [14]. This latter study also tested by CFA a single factor model where all items loaded into one overall self-compassion factor, but failed to find an acceptable fit. Results are more consistent regarding the scale’s reliability, with studies demonstrating a high internal consistency of the total scale, and sufficient to good internal consistency of the subscales among college students [7,15]. Finally, regarding the convergent validity, the SCS total score has been related to measures of self-esteem, neuroticism, and rumination, considered to be theoretically related constructs. In student samples, a strong positive relationship has been observed with self-esteem as well as strong negative relationships with rumination and neuroticism [7,10]. Also, there is evidence that Buddhist practitioners of Vipassana mediation report more self-compassion than college students [7]. However, the large demographic differences between these two groups prevent drawing a firm conclusion on this finding.

Given that the SCS was developed using a student sample and its psychometric properties have been commonly tested in college students, we conducted a study among community adults to test its psychometric qualities. Firstly, we examined the factor structure of the SCS using CFA, testing whether we could replicate the (hierarchical) six-factor structure, as suggested by Neff [7]. In case the CFA failed to support the six-factor structure, we intended to perform an exploratory factor analysis (EFA) to explore the SCS factorial structure. Secondly, we evaluated its reliability in terms of internal consistency. Thirdly, to explore the scale’s construct validity, we examined the relationships between the SCS and self-report measures of positive and negative affect, depressive symptoms, and perceived stress, as well as with the assumed theoretically related constructs of rumination and neuroticism.

## Method

### Sample and procedure

This study is part of a research on quality of life, mindfulness and related constructs including self-compassion. This research was approved by the medical ethical committee of the University Medical Center of Groningen, The Netherlands. A community-based sample was selected from the register offices of five municipalities in The Netherlands, based on the age and gender distribution of the Dutch general population (in the time the data was collected 50.5% of the Dutch total population was female, 33% was 20 to 40 years old, 47% was 41 to 65 years old, 15% was 66 to 80 years old, and 5% was 81 or more years old). When having obtained the names and addresses from the municipalities, people were sent a letter with brief information about the focus of the study and with the invitation to participate by filling in a self-report questionnaire. They were also sent an informed consent, the self-report questionnaire package and a return envelope, so they could return the informed consent and questionnaire without any costs. A total of 7492 persons were approached of whom 24.4% agreed to participate and sent back the informed consent and questionnaire package. Participants that failed to complete 15% or more of the questionnaire package were excluded. A total of 1736 adults constituted the final sample. For the present study, cases with missing values on any of the SCS’s items were excluded. Analyses were then performed with a sample of 1643 adults, 54.8% female, and 45.2% male. Participants’ mean age was 54.9 years old (*SD* = 16.7), ranging from 20 to 97 years old (22% was 20 to 40 years old, 50% was 41 to 65 years old, 21% was 66 to 80 years old, and 7% was 81 or more years old). A 19.5% of the sample was low educated, 48.7% middle educated and 31.8% high educated. The majority of the sample was married or cohabiting (76.6%), followed by single (9.5%), widowed (6.9%), divorced (4.1%), and other (2.8%). The sample was primarily employed (50.1%), 24.1% was retired, 10.4% did housework, 3.8% did volunteer work, 3.4% was on disability, and 8.4% did other activities.

### Measures

#### Self-compassion

The 24-item Dutch version of the Self-Compassion Scale (SCS) [7,16] was used. Neff and Vonk [16] translated the original SCS into Dutch, removing two of the 26 items from the original English version due to difficulties in translation (the authors did not mention in the study which type of difficulties they encountered with the translation of these two items). The SCS is divided into six subscales: 4-item Self-Kindness (e.g., ‘I am kind to myself when I am experiencing suffering’); 4-item Self-Judgment (e.g. ‘I am intolerant and impatient towards those aspects of my personality I don’t like’); 4-item Common Humanity (e.g. ‘I try to see my failings as part of the human condition’); 4-item Isolation (e.g., ‘When I think about my inadequacies it tends to make me feel more separate and cut off from the rest of the world’); 4-item Mindfulness (e.g., ‘When I fail at something important to me I try to keep things in perspective’); and 4-item Over-Identification (e.g., ‘When I fail at something important to me, I become consumed by feelings of inadequacy’). The items can be rated on a five-point likert scale with 1 indicating *almost never* and 5 indicating *almost always*. After reversing the negatively formulated items, a total score can be calculated, which may range from 24 to 120, with higher scores indicating greater self-compassion.

#### Positive and Negative Affect

Positive and negative affect were measured with the 20-item Positive and Negative Affect Schedule (PANAS) [17,18]. This instrument is divided into two 10-item scales that assess feelings of activeness, enthusiasm, and alertness (i.e., positive affect), and subjective distress and unpleasant engagement (i.e., negative affect). Participants were asked to rate the extent to which they experienced each particular emotion during the last week using a five-point likert scale (1 indicating *very slightly or not at all* and 5 indicating *very much*). Total scores are calculated for each scale by summing all the 10 items and can range from 10 to 50. Higher scores indicate more positive and negative affect. The PANAS has demonstrated good internal consistency for the positive affect and negative affect scales [19]. In this study, the positive affect and negative affect scales of the PANAS showed good internal consistency (α = .88 and .87, respectively).

#### Depressive symptoms

Depressive symptoms were assessed with the Center of Epidemiologic Studies Depression Scale (CES-D) [20–22]. The CES-D is a 20-item self-report instrument designed to measure current levels of depressive symptomatology in the general population. The scale consists of 16 negatively formulated items (e.g., ‘I felt depressed’) and four positively formulated items (e.g., ‘I enjoyed life’). On a four-point likert scale, participants specified the frequency by which each symptom was experienced during the last week (0 indicating *rarely or none of the time* and 3 indicating *most of the time*). After reversing the positively formulated items, a total score can be calculated based on all 20 items. Total scores may range from 0 to 60, with higher scores indicating more depressive symptoms. The CES-D has shown a good internal consistency [20]. Similarly, in this study the scale’s internal consistency was good (α = .89).

#### Perceived stress

The 4-item version of the Perceived Stress Scale (PSS) [23,24] was used to measure experiences of stress. Participants rated in a five-point likert scale, ranging from 0 (*never*) to 4 (*very often*), the frequency on which they experienced stress during the last month (e.g. ‘In the last month, how often have you felt that you were unable to control the important things in your life?’). After reversing the positively formulated items, a total score can be calculated based on the four items. Total scores can range from 0 to 16, with higher scores representing greater levels of perceived stress. This version of the scale has demonstrated acceptable internal consistency and adequate test-retest reliability over a 2-month period [23]. The PSS had an acceptable internal consistency (α = .73) in the current study.

#### Neuroticism

Neuroticism was measured with the 12-item neuroticism scale of the NEO Five-Factor Inventory (NEO-FFI) [25,26]. Participants rated the extent to which they agreed with 12 statements (e.g., ‘I am not a worrier’) using a five-point likert scale (1 indicating *strongly disagree* and 5 indicating *strongly agree*). After reversing the positively formulated items, a total score can be calculated based on the 12 items. Higher scores are indicative of greater neuroticism, with total scores ranging from 12 to 60. Costa & McCrae [25] found good internal consistency and adequate test-retest reliability over a 3-month interval range. This scale showed good internal consistency in the current study (α = .87).

#### Rumination

The 12-item rumination subscale of the Rumination-Reflection Questionnaire (RRQ) [27,28] was used to assess rumination. Participants indicated on a five-point likert scale ranging from 1 (*strongly disagree*) to 5 (*strongly agree*), the extent to which they involved in ruminative thinking (e.g., ‘I spend a great deal of time thinking back about my embarrassing or disappointing moments’). A total score can be calculated by reversing the positively formulated items and summing the 12 items. Total scores can range from 12 to 60. Higher scores indicate greater levels of rumination. Trapnell & Campbell [27] reported excellent internal consistency. This scale showed good internal consistency in the present study (α = .89).

### Data analysis

The factor structure of the SCS was first tested with CFA using weighted least squares method based on polychoric correlation matrix. Analyses were performed in MPlus, version 7.1 [29]. The goodness of fit of the models was evaluated using the chi-squared to degrees of freedom ratio (*χ*2/*df*), the comparative fit index (CFI), the tucker-lewis index (TLI), the root mean square error of approximation (RMSEA), and the weighted root mean residual (WRMR). The *χ*2/*df* values close to or less than 2, and less than 5, were interpreted as indicative of good and acceptable fit of the model, respectively [30]. CFI and TLI values ≥ .90 and ≥.95, respectively, were considered to show acceptable and good model fit. RMSEA values ≤.06 were considered as an indication of good fit, and in the range of .06 to .08 were considered to indicate an acceptable model fit [31]. WRMR values ≤1.0 were considered to indicate a good fit [32]. Giving that these fit indices can be influenced by sample size and data normality [33], a satisfactory model fit was interpreted when all indices met either an acceptable or a good fit.

A series of EFA were conducted in SPSS 20.0 to further examine the factor structure of the SCS. Maximum likelihood method with varimax rotation was used since the objective was to identify latent underlying constructs and there were not assumptions of the factors as being related. Following suggestions of Fabrigar et al. [34] regarding EFA, the distribution of the items was examined to ensure there were not severe nonnormalities (skewness > 2; kurtosis > 7). None of the SCS’s items showed severe nonnormal distribution. The number of relevant factors was determined based on the scree plot and using the minimum average partial (MAP) statistical test [35,36]. The MAP uses the remaining variance of the correlation matrix after component’s extraction as criterion for determining the number of relevant factors. Research suggest that the MAP performs superior than commonly used rules of thumb such as the eigenvalues-greater-than-one rule [37] which tends to overestimate the number of factors to extract [38,39]. Due to the large sample size, loadings above .20 were considered significant [40]. Internal consistency was analysed with Cronbach’s alpha statistic. Values of .80 or higher were considered as good [41]. Pearson correlations were used to test the associations between the SCS and other measures of psychological functioning; correlations coefficients below 0.3 were interpreted as small or weak, from 0.3 to 0.5 as moderate and above 0.5 as strong [42].

## Results

### Descriptive Statistics

The mean scores and standard deviations (*SD*) of all study variables are presented in Table 1. The inter-correlations among the original SCS’s six subscales and their correlations with the SCS total score are presented in Table 2.

**Table 1:** Means (and *SD*s) of study variables.

Variable (scale)	*M* (*SD*)
Self-compassion (SCS)	80.13 (12.75)
Negative affect (PANAS-NA)	15.66 (5.89)
Depressive symptoms (CES-D)	9.33 (8.27)
Perceived stress (PSS)	4.55 (2.70)
Positive affect (PANAS-PA)	30.25 (7.32)
Neuroticism (NEO-FFI-Neuro)	28.96 (8.00)
Rumination (RRQ-Rumin)	35.60 (8.30)

SCS = Self-Compassion Scale; PANAS-NA = Positive and Negative Affect Schedule-Negative Affect scale; CES-D = Center of Epidemiologic Studies Depression Scale; PSS = Perceived Stress Scale; PANAS-PA = Positive and Negative Affect Schedule-Positive Affect scale; NEO-FFI-Neuro = NEO Five-Factors Inventory-Neuroticism subscale; RRQ-Rumin = Rumination Reflection Questionnaire-Rumination subscale.

**Table 2:** Inter-correlations between the SCS’s subscales and correlations with the SCS total score.

	SK	CH	M	SJ	I	SCS total score
**SK**	-					.64***
**CH**	.53***	-				.47***
**M**	.62***	.55***	-			.65***
**SJ**	-.18***	.08**	-.06*	-		-.61***
**I**	-.14***	.02	-.18***	.62***	-	-.70***
**OI**	-.11***	.06*	-.17***	.60***	.74***	-.67***

* *p* < .05

** *p* < .01

*** *p* < .001

SK = Self-Kindness; CH = Common Humanity; M = Mindfulness; SJ = Self-Judgment; I = Isolation; OI = Over-Identification; SCS = Self-Compassion Scale.

### Confirmatory and exploratory factor analysis

Following Neff [7], a six-factor hierarchical model was tested but it could not be estimated due to weak correlations between some pairs of the six factors that prevented a higher-order factor from emerging. Subsequently, a model with six correlated factors was tested. The fit indices indicated that the model did not fit the data sufficiently: *χ*2 = 3779.467 (*df* = 237, *p* < .000), *χ*2/*df* = 15.95 (i.e., > 5 indicating a non-acceptable fit), CFI = 0.896 (i.e., < .90 indicating a non-acceptable fit), TLI = 0.879 (i.e., < .90 indicating a non-acceptable fit), RMSEA = 0.095 (i.e., > .08 indicating a non-acceptable fit), WRMR = 3.146 (i.e., > 1 indicating a non-good fit).

Giving that the six-factor structure could not be replicated, an EFA was subsequently conducted. The scree plot and MAP suggested the presence of two relevant factors. Therefore, a second EFA was conducted with two fixed factors. With an eigenvalue of 6.35, Factor 1 explained 26.5% of the variance. Factor 2 had an eigenvalue of 4.53 and explained 18.9% of the variance. After rotation, the 12 negatively formulated items loaded on factor 1 (i.e., negative factor) and the 12 positively formulated items loaded on factor 2 (i.e., positive factor). The total explained variance of this two-factor solution was 45.4%, with the percentage of unexplained variance being attributed to the high heterogeneity of the SCS’s items. The factor loadings for each item on the two factors before and after rotation are presented in Table 3.

**Table 3:** Item-factor loadings before and after rotation for exploratory factor analysis.

Item description and subscale		Item formulation	Non-rotated		Rotated	
			Factor 1	Factor 2	Factor 1	Factor 2
Feel alone in my failure	I	Neg	.78	-.23	**.81**	.05
Obsess and fixate on everything that’s wrong	OI	Neg	.77	-.19	**.79**	.08
Get down on myself	SJ	Neg	.76	-.19	**.78**	.08
Feel like others are probably happier	I	Neg	.70	-.16	**.71**	.09
Get carried away with my feelings	OI	Neg	.64	-.20	**.67**	.03
Feel like others must be having an easier time	I	Neg	.65	-.16	**.67**	.07
Feel separate and cut off from the world	I	Neg	.66	-.09	**.65**	.14
Judgmental about my own flaws	SJ	Neg	.61	-.20	**.64**	.02
Intolerant towards aspects that I don’t like	SJ	Neg	.61	-.18	**.64**	.04
Consumed by feelings of inadequacy	OI	Neg	.60	-.09	**.63**	.01
Blow the incident out of proportion	OI	Neg	.47	-.25	**.47**	.08
To be tough on myself	SJ	Neg	.23	-.25	**.30**	-.15
See my failings as part of the human condition	CH	Pos	.36	.64	.13	**.73**
Keep things in perspective	M	Pos	.29	.63	.06	**.69**
Balanced view of the situation	M	Pos	.32	.59	.11	**.66**
Keep my emotions in balance	M	Pos	.34	.56	.13	**.64**
Understanding towards aspects I don’t like	SK	Pos	.23	.57	.02	**.62**
Tolerant of my own flaws	SK	Pos	.32	.52	.16	**.61**
Feelings of inadequacy shared by most people	CH	Pos	.19	.57	-.02	**.60**
Approach feelings with curiosity	M	Pos	.21	.54	.02	**.58**
Give to myself caring and tenderness	SK	Pos	.39	.45	.21	**.56**
Difficulties part of life everyone goes through	CH	Pos	.04	.53	-.14	**.51**
I’m kind to myself	SK	Pos	.18	.46	.01	**.50**
Other people in the world feeling like me	CH	Pos	-.09	.43	-.23	**.38**

Negative formulated items were reversed for EFA. I = Isolation, OI = Over-Identification, SJ = Self-Judgment, CH = Common Humanity, M = Mindfulness, SK = Self-Kindness.

Two additional EFA were conducted to examine the structure of these two factors in order to explore whether within each factor we could find the three components of self-compassion as suggested by Neff [7]. That is, self-kindness, common humanity and mindfulness within the positive factor, and self-judgment, isolation and over-identification within the negative factor. The scree plots and MAP suggested one-factor solutions for both factors. The two identified factors were weakly negatively correlated (*r* = -.11, *p* < .001). The following analyses examine the reliability and validity of these two factors. Although CFA and EFA results did not support the use of the SCS total score, its reliability and validity are also reported as complementary information giving that most past and current research is using this total score.

### Reliability: Internal consistency

The positive and negative factors (i.e., the sum scores of the 12 positively and 12 negatively formulated items) showed good internal consistency, with Cronbach’s alpha coefficients of .86 and .90, respectively. The SCS total score demonstrated good internal consistency with a Cronbach’s alpha coefficient equal to .86.

### Construct validity: Correlations with other self-report measures

#### Relationship of SCS to indicators of psychological functioning

The negative factor was moderately to strongly positively related to negative affect, depressive symptoms, and perceived stress, and weakly negatively related to positive affect. The positive factor, on the other hand, showed small negative correlations with negative affect, depressive symptoms, and perceived stress, and a small positive correlation with positive affect relatively stronger than the one of the negative factor. Similarly to the negative factor, the SCS total score had moderate to strong negative correlations with measures of negative affect, depressive symptoms, and perceived stress, and a small positive correlation with positive affect (Table 4).

**Table 4:** Correlations between the SCS total score, SCS positive factor and SCS negative factor with self-report measures of psychological functioning, neuroticism and rumination.

Variable (scale)	SCS total score	SCS Positive factor	SCS Negative factor
**Negative affect (PANAS-NA)**	-.47***	-.15***	.52***
**Depressive symptoms (CES-D)**	-.53***	-.26***	.52***
**Perceived stress (PSS)**	-.53***	-.29***	.48***
**Positive affect (PANAS-PA)**	.26***	.29***	-.11***
**Neuroticism (NEO-FFI-Neuro)**	-.69***	-.33***	.68***
**Rumination (RRQ-Rumin)**	-.57***	-.17***	.65***

*** *p* < .001

SCS = Self-Compassion Scale; PANAS-NA = Positive and Negative Affect Schedule-Negative Affect scale; CES-D = Center of Epidemiologic Studies Depression Scale; PSS = Perceived Stress Scale; PANAS-PA = Positive and Negative Affect Schedule-Positive Affect scale; NEO-FFI-Neuro = NEO Five-Factors Inventory-Neuroticism subscale; RRQ-Rumin = Rumination Reflection Questionnaire-Rumination subscale.

#### Relationship of SCS to neuroticism and rumination

The negative factor showed a strong positive correlation with neuroticism and rumination. In contrast, the positive factor showed a moderate negative correlation with neuroticism and a small negative correlation with rumination. The SCS total score showed a strong negative correlation with these constructs (Table 4).

## Discussion

This study examined the psychometric properties of the Self-Compassion Scale in a large community sample. Results did not confirm the (hierarchical) six-factor structure, as proposed by Neff [7]. In contrast, two factors were found, formed by the positively and negatively formulated items. The internal consistency of these two factors was good. Importantly, the two factors showed different patterns of correlations with other measures, suggesting a different meaning for both factors. The negative factor was moderately to strongly related to psychological symptoms, rumination and neuroticism. In contrast, the positive factor was only weakly to moderately related to psychological symptoms, rumination, and neuroticism, and relatively more strongly related to positive affect.

A key finding is that we could not confirm the suggested six-factor structure for the SCS, with results indicating two factors based on item formulation (i.e., positive or negative). Our results are in line with those of others, who could also not adequately replicate the assumed six-factor structure [14]. In the original study [7], the SCS’s factor structure was examined by conducting separate CFA for each of the three suggested components of self-compassion, with results showing two distinct factors within each component, based on item formulation: self-kindness versus self-judgment, common humanity versus isolation, and mindfulness versus over-identification. When analysing all items simultaneously (rather than for each component separately), we found that the positively and negatively formulated items formed separated factors, indicating that self-kindness, common humanity and mindfulness combine into one factor, and self-judgment, isolation, and over-identification into another factor. The commonality between the results of Neff [7] and our results is the role of item formulation in differentiating distinct factors.

It can be reasoned that the two found factors are caused by an artificial method effect as previous research has suggested this can be the case when two factors are solely composed by positively and negatively formulated items [43,44]. Alternatively, it can be reasoned that it is theoretically accurate to separate the SCS’s positive and negative items since their content seem to be measuring two different processes: self-compassion and self-criticism, instead of one construct of self-compassion. Supporting this idea, Gilbert et al. [45] argued that self-compassion is distinct from self-criticism, related to different affective and physiological systems, and therefore they should not be measured as one. The inclusion of both positive and negative items in the SCS suggests that self-compassion is conceptualized as a bipolar construct, ranging from high self-compassion (as measured by the positive items) to high self-criticism (as measured by the negative items). However, there is evidence supporting the distinction between self-compassion and self-criticism as independent processes [46], and more broadly between positive, resilience factors and negative, vulnerability factors [47]. Using a fMRI task, Longe et al. [46] found that self-critical thinking was associated with regions of the brain related to error processing/resolution and behavioral inhibition, while self-reassurance was associated to regions that are also activated when expressing compassion and empathy towards others. These results suggest that the neural correlates of self-compassion and self-criticism are different, supporting their view as independent processes.

Further evidence for the differential meaning of the positive and negative factors was the distinct pattern of correlations with other measures. The negative factor was strongly related to rumination and neuroticism, while the positive factor related moderately to neuroticism and weakly to rumination. The strong correlations of the negative factor with these constructs might be explained by the fact that its items seem to be measuring self-criticism. Previous research suggests that both rumination and neuroticism are strongly related to self-criticism [48–50]. Moreover, a construct overlap between neuroticism and self-criticism was proposed [50], with latter evidence supporting their view as independent but highly related constructs [51]. In addition, self-criticism has shown to be an important prospective predictor of depression [52,53]. Accordingly, our results showed a strong relationship between the negative items of the SCS (i.e., negative factor) and depressive symptoms, a finding in line with others [54,55].

The different pattern of correlations between the two found factors and measures of wellbeing, might further suggest a difference in their role for predicting wellbeing. The stronger correlations of the negative factor with depressive symptoms, negative affect and perceived stress, support the view of self-criticism as an important vulnerability factor for the experience of psychological symptoms [52,53]. In turn, the stronger correlation of the positive factor with positive affect might suggest that self-compassion is a protective, resilience factor. These findings highlight the importance of distinguishing self-compassion from self-criticism when examining its predictive role on wellbeing.

Our findings have some implications for clinical settings. As we could not confirmed a hierarchical six-factor structure for the SCS, we prevent clinicians from using a SCS total score as an indicator of self-compassion. Moreover, due to the inclusion of positively and negatively formulated items, a SCS total score does not differentiate between levels of self-compassion and self-criticism. Our results suggest different correlates for the two found factors, thus, we advise to separately use the SCS’s positive and negative items. More research is needed to confirm our findings and the validity of the two factors.

When interpreting our findings, some limitations need to be considered. This study focused on the factor structure of the SCS, with a limited amount of variables available to examine convergent validity and none to examine discriminant validity. In addition, the criterion validity of the scale was not examined giving the fact that the SCS is currently the only available self-report measure of self-compassion. The sample was recruited by mail, with a response rate of 24.4%. Unfortunately, information about the socio-demographics of the nonresponding sample was not available to check for possible selection bias; however, the gender and age distribution of the studied sample was similar to those of the general Dutch population. Our response rate is not uncommon for mail surveys [56], but it can be argued that the topic of the study (i.e., quality of life, mindfulness and related constructs) may have reduced the participation considering that responders were a large group from the general population not particularly motivated or interested in this topic. In addition, the length of the questionnaire package (about 30 minutes to fill in) may have reduced the response rate. Our findings may not be generalizable to clinical populations giving that we focused on a community sample. Evidence suggests differences between these populations in levels and correlates of self-compassion [57]. However, a recent study did not find differences between depressed patients and adults from the community regarding the factor structure of the SCS [14].

To conclude, our results suggest that it is meaningful to distinguish self-compassion from harsh self-criticism and do not support the use of a SCS total score as a measure of self-compassion. Considering the rapid increase of research on self-compassion, we strongly encourage a continued psychometric assessment of the SCS, in both nonclinical and clinical populations, particularly the replication of the two-factor structure.
